# The role of structured education in the management of hypoglycaemia

**DOI:** 10.1007/s00125-017-4334-z

**Published:** 2017-06-28

**Authors:** Ahmed Iqbal, Simon R. Heller

**Affiliations:** Department of Oncology and Metabolism, Room EU38, E Floor, School of Medicine and Biomedical Sciences, University of Sheffield, Beech Hill Road, Sheffield, S10 2RX UK

**Keywords:** Hypoglycaemia, Review, Structured education, Type 1 diabetes, Type 2 diabetes

## Abstract

The role of intensive glycaemic control in preventing microvascular disease in diabetes is well established. Iatrogenic hypoglycaemia is, however, a major barrier to effective treatment. Hypoglycaemia is associated with a significant level of morbidity and, despite pharmacological and technological therapeutic advances, reported rates of severe hypoglycaemia in clinical practice have not fallen over the last 20 years. This suggests that human factors are of major relevance and that ensuring the effective self-management of diabetes is an important strategy for the reduction of hypoglycaemic risk. Most of the evidence for the impact of this strategy on hypoglycaemia risk is confined to adults with type 1 diabetes although, in this review, we also cite studies that have specifically addressed this in type 2 diabetes. There are relatively few adequately powered RCTs that have rigorously evaluated the effectiveness of structured education and training programmes on hypoglycaemia; however, the available data suggest a subsequent reduction in severe hypoglycaemia rates of around 50%, a rate reduction that is comparable with that observed following technological interventions. Furthermore, longitudinal observational cohorts support these data, showing similar reductions in rates of hypoglycaemia following structured education. Those who continue to experience recurrent hypoglycaemia and impaired awareness of hypoglycaemia despite education and training in diabetes self-management may benefit from technological interventions and/or interventions that specifically address psychological factors that contribute to hypoglycaemia risk; however, there is urgent need for further research in this area. In the meantime, structured education for effective self-management of diabetes should be part of routine therapy for all those with type 1 diabetes.

**Electronic supplementary material:**

The online version of this article (doi:10.1007/s00125-017-4334-z) contains a slide of the figure for download, which is available to authorised users.

## Introduction

The importance of intensive glycaemic control in preventing complications in both type 1 and type 2 diabetes is well established [1, 2]. However, these early studies also demonstrate that intensive glucose control increases the risk of severe hypoglycaemia [1, 2], although recent clinical trials suggest that the overall risk of severe hypoglycaemia is now falling substantially using current glucose-management strategies [3, 4]. This is in marked contrast with observational studies [5–7] that show that severe hypoglycaemia rates remain comparable with those reported over 20 years ago [8], despite the introduction of insulin analogues, continuous subcutaneous insulin infusion (CSII) and continuous glucose monitoring (CGM). These observations indicate that additional factors contribute to the risk of hypoglycaemia in clinical practice. In this review, we argue that effective self-management is the key to reaching glucose targets while minimising the risk of hypoglycaemia.

Here, we will first briefly consider the definition and epidemiology of hypoglycaemia in diabetes. As a disease, type 1 diabetes is arguably unique, given the relentless demand it places on an individual to self-manage their condition. Since education and training are likely to be critical for acquiring self-management skills, we will focus on the role of structured education/training in the clinical management of type 1 diabetes. In referring to structured education, we cite literature where a fixed curriculum has been used by trained educators to teach individuals to self-manage their diabetes more effectively. When describing the various methods used to evaluate the effects of the education/training programmes on hypoglycaemia, we highlight important differences that we consider to be relevant. Psychological factors are equally important in determining a person’s ability to effectively self-manage their condition and while a detailed review of studies addressing these factors is beyond the scope of this review, inevitably, high-quality structured education addresses some psychological factors, at least in part.

For this review, we searched PubMed (MEDLINE) and EMBASE, from inception (1964 and 1974, respectively) until 1 October 2016, using these keywords and synonyms in addition to ‘self’ and ‘management’ and ‘self-management’: ‘hypoglycaemia’; ‘low glucose’; ‘hypoglycaemia avoidance’, ‘structured’ and ‘education’; ‘structured-education’, ‘insulin’ and ‘training’; ‘insulin-training’; ‘carbohydrate’ and ‘counting’; ‘carbohydrate-counting’. We additionally used a recently published systematic review and meta-analysis [9] to reconcile studies on type 1 diabetes that were not identified in our original search.

## Epidemiology of hypoglycaemia

An ADA working group [10] defined symptomatic hypoglycaemia as the presence of typical symptoms of hypoglycaemia with a glucose measurement <3.9 mmol/l and severe hypoglycaemia as any episode requiring the assistance of another person to recover. These definitions have limitations, however, and universal application is difficult. For example, paediatricians generally include hypoglycaemic episodes that cause coma or seizures in their definition for severe hypoglycaemia (since young children always need external assistance). Furthermore, many studies report hypoglycaemic episodes at a glucose level lower than 3.9 mmol/l, often on the basis that they are more clinically relevant. This inconsistency in hypoglycaemia classification makes it difficult to compare different studies and interventions. In particular, episodes of hypoglycaemia that may lead to impaired awareness of hypoglycaemia (IAH) or that are associated with mortality are not recorded systematically according to an agreed classification.

Recently, the ADA and the EASD agreed a position statement that proposes the inclusion of a third glucose level, denoting ‘clinically important’ (in addition to <3.9 mmol/l and severe hypoglycaemia) at <3.0 mmol/l for the classification of hypoglycaemia [11]. If this classification was universally adopted for use in clinical trials, it could enable reliable comparisons of the effectiveness of educational or therapeutic interventions for the reduction of hypoglycaemia rates in diabetes. However, the new proposed criteria for hypoglycaemia set by the ADA/EASD have yet to be agreed more widely. Thus, in this review we will generally confine our comments to severe hypoglycaemia in adults, the definition of which (severe hypoglycaemia: requiring assistance of another person to recover) is currently widely accepted.

## Studies of severe hypoglycaemia in adults with diabetes

Most studies reporting severe episodes of hypoglycaemia in diabetes are associated with several limitations. First, the data are generally compiled from patient-reported episodes and since retrospective studies rely on an individual’s recall of events, they are susceptible to recall bias. Somewhat arbitrarily, the frequency of non-severe hypoglycaemic episodes is considered reliable if the data are collected retrospectively at monthly intervals, while severe episodes are thought to be accurately identified for up to 1 year later. Episodes collected prospectively are likely to be more reliable in ascertaining the true frequency and predictors of hypoglycaemia, although, since data are collected over relatively brief periods (4–12 weeks) and then extrapolated to establish annual incidence, this may introduce sampling error. Second, studies that seek recruits by advertising may exhibit selection bias since individuals with diabetes with hypoglycaemic problems are arguably more likely to participate. Few studies are truly population based because of the challenge of identifying all individuals with diabetes. Third, rates of hypoglycaemia among people with diabetes are not normally distributed, with many individuals reporting no events and a few experiencing a high number of events; statistical analyses can adjust for this but are not always applied. Fourth, the reporting of hypoglycaemia is particularly unreliable in elderly individuals, in whom the symptomology of hypoglycaemia is different from that in younger people. In elderly people, episodes of hypoglycaemia (even those that are severe) may be attributed to cerebrovascular events [12]. Finally, many studies recruit relatively small numbers and are therefore underpowered to compare the effects of therapeutic interventions (including educational interventions) on rates of severe hypoglycaemia.

One of the most reliable assessments of hypoglycaemic burden has emerged from a true population-based prospective study. In this study, the annual incidence of severe hypoglycaemia episodes in insulin-treated type 2 diabetes was observed to be less than half of that in type 1 diabetes (1.15 events per person per year in type 1 diabetes vs 0.35 events per person per year in type 2 diabetes) [13]. In both type 1 and type 2 diabetes, less than 10% of the study population had a mean HbA_1c_ <7% (53 mmol/mol) and HbA_1c_ was not a significant predictor of prospective hypoglycaemia (*p* = 0.248 and *p* = 0.099 for type 1 and type 2 diabetes, respectively).

The prevalence of IAH is estimated to be 50% in those with type 1 diabetes after 25 years or more of treatment [14–16]. In insulin-treated type 2 diabetes, in one observational study, the prevalence of IAH was reported to be ~10%, although confirmatory data are lacking [17]. Duration of treatment with insulin is a key predictor of the risk of hypoglycaemia, with rates of severe episodes rising in both type 1 and type 2 diabetes with longer treatment duration [5]. Interestingly, hypoglycaemia risk in type 1 and type 2 diabetes was comparable in one study in which participants were matched for duration of treatment with insulin [18]. A global rise in the incidence of type 2 diabetes [19] coupled with improved access to insulin treatment and longer life expectancies means that the management of hypoglycaemia in type 2 diabetes is becoming increasingly important. In numerical terms, at least, the burden of hypoglycaemia as a clinical problem is already greater in type 2 diabetes than in type 1 diabetes.

## Structured education programmes in the clinical management of hypoglycaemia

Programmes aimed at reversing IAH and reducing the risk of severe hypoglycaemia are based on the premise that reduced neuroendocrine and symptomatic responses to hypoglycaemia, even in long-standing IAH, can be reversed with meticulous avoidance of further hypoglycaemia [20, 21]. However, the potential for structured education/training to reduce hypoglycaemia emerged many years before the research that first identified the contribution of antecedent hypoglycaemia to reduced awareness [22, 23].

## The Diabetes Teaching and Treatment programme

In Düsseldorf, Germany, Muhlhauser and colleagues recognised the opportunity that the introduction of self-monitoring of blood glucose (SMBG) presented in improving management of type 1 diabetes. The fundamental principles of the Diabetes Teaching and Treatment programme (DTTP) included separating basal and pre-meal bolus insulin, intensive SMBG (including nocturnal testing), counting carbohydrates to permit a flexible diet that is comparable with that of people without diabetes, and a structured written curriculum delivered by trained educators. The course was delivered over 5 days to inpatients in groups of six to eight.

The DTTP was evaluated in a series of clinical trials conducted in countries behind the Iron Curtain, since the authors considered that the spreading influence of the DTTP programme had ‘contaminated’ German diabetes centres. Having established that the course resulted in markedly improved HbA_1c_ and fewer hospital admissions [24], the authors went on to conduct a large controlled trial in 300 participants [25]. Outcomes in those undertaking the course were compared at 24 month follow-up with a waiting-list control group and also with a control group taught basic information. Participants were taught to calculate insulin doses based on the carbohydrate value of different meals, while keeping blood glucose within tight targets. The course resulted in a long-term improvement in glycaemic control (HbA_1c_ was improved by 1.5% at 1 year follow-up compared with control participants taught basic information alone), which was accompanied by a marked reduction in ketoacidosis, although hypoglycaemia rates were unchanged. In another DTTP trial, Starostina et al [26] reported that mean HbA_1c_ was reduced by 3% in the intervention group compared with the control group at 1 year of follow-up, again with no reported difference in rates of severe hypoglycaemia. These studies, though promising, are limited by being underpowered to demonstrate differences in rates of severe hypoglycaemia.

**Fig. 1:**
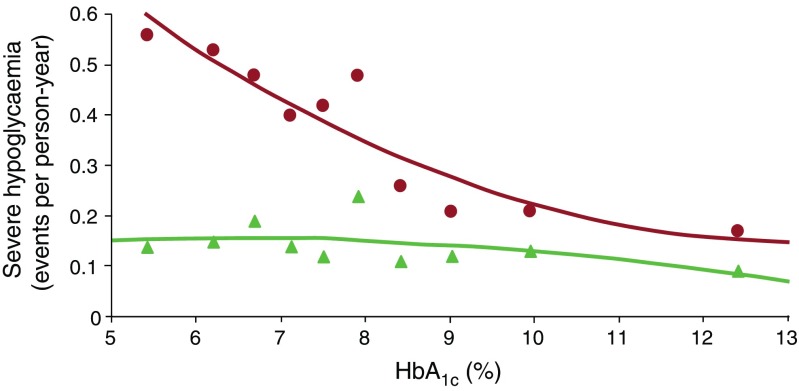
Evaluation of the efficacy of teaching flexible, intensive insulin therapy as part of a structured training course on glycaemic control and severe hypoglycaemia rates in 9583 individuals with type 1 diabetes between 1992 and 2004. Participants underwent 20 h of training as inpatients and were advised to measure blood glucose before main meals and at bedtime. Insulin was adjusted to actual blood glucose level and intended carbohydrate intake. Red circles, 1 year prior to intervention; green triangles, 1 year after intervention [28]. To convert values for HbA_1c_ in DCCT % into mmol/mol, subtract 2.15 and multiply by 10.929. Adapted from [28] with permission of Springer-Verlag

A large observational study from the same group [27] presented findings from a 6 year follow-up in a cohort of individuals with type 1 diabetes who had undergone the DTTP. They reported a reduction in HbA_1c_ of 0.7%, while the incidence of severe hypoglycaemia had fallen from 0.28 episodes per person per year in the year before DTTP to 0.17 episodes per person per year at follow-up (*p* < 0.05). Furthermore, another observational study demonstrated that the exponential relationship between the level of HbA_1c_ and the risk of severe hypoglycaemia (which was clearly present before participation in the course) could be abolished 12 months following inpatient DTTP training (Fig. 1) [28]. Although observational studies provide a weaker level of evidence compared with clinical trials, the large number of participants and the long period of follow-up strongly suggest that participation in the DTTP improves HbA_1c_ while reducing severe hypoglycaemia by approximately 50% [28, 29].

## DTTP-based programmes: ‘dose adjustment for normal eating’ and ‘Tayside insulin management’

The reported success of these programmes has led to their adoption across a range of countries. In the UK, a multicentre RCT measured the impact of a structured education course (Dose Adjustment for Normal Eating [DAFNE]), modelled on the DTTP, on glycaemic control and quality of life in type 1 diabetes [30]. At three UK centres, 169 individuals with moderate-to-poor glycaemic control (HbA_1c_ 7.5–12% [58.5–107.7 mmol/mol]) participated in a 5 day structured education outpatient programme taught by two educators (diabetes specialist nurses and dietitians). Participants were randomised either to attend the DAFNE course immediately or to receive usual care for 6 months prior to attending the course, thus acting as a waiting-list control group. There were modest improvements in HbA_1c_ (0.7–1% at 6 months; *p* < 0.0001), whilst no change was observed in the control group. However, there was not a significant reduction in the incidence of severe hypoglycaemia, perhaps because the trial was underpowered to detect a difference.

Importantly, longer-term observational studies involving larger numbers of participants have demonstrated reductions in the incidence of severe hypoglycaemia that are comparable with those seen in the original DTTP studies from Germany, albeit at higher HbA_1c_ levels. These studies report reductions in HbA_1c_ (mean difference pre- and post-DAFNE: 0.27%; *p* < 0.001) and around a 50% reduction in rates of severe hypoglycaemia (mean ± SD: pre-DAFNE 1.7 ± 8.5 vs post-DAFNE 0.6 ± 3.7 episodes per person per year; *p* < 0.05), together with improved awareness of hypoglycaemia in up to 43% of participants at 1 year follow-up [31]. The overall rate of IAH fell from 39.9% to 33%, with reductions in psychological distress and improved wellbeing up to 1 year following programme attendance [31].

Similarly, observational data from a study of the Tayside insulin management (TIM) structured education programme showed a reduction in HbA_1c_ (mean 0.4%; *p* < 0.001) following 6 months of participation, a reduction in the annual incidence of severe hypoglycaemia requiring parenteral treatment and improved awareness of hypoglycaemia in 25% of participants [32].

Of note, in both the DAFNE and TIM study, IAH was not confirmed using an objective measure (such as the Gold score). However, the DAFNE authors did establish a robust definition for IAH in the form of a hypoglycaemia awareness rating that may be less subjective than the Gold score [31, 33].

## Programmes focussing on improving hypoglycaemia awareness

### The Recovery of Hypoglycemia Awareness in Long-Standing Type 1 Diabetes (HypoCOMPaSS)

The HypoCOMPaSS trial seems to suggest that even a brief educational intervention focussed on hypoglycaemia detection in type 1 diabetes can produce a significant improvement in the rate of moderate and severe hypoglycaemia without compromising metabolic control [34]. The trial involved 96 adults with type 1 diabetes and IAH, randomised in a 2 × 2 factorial design to CGM, insulin pumps or multiple daily injections alone. All participants received a brief half-day education programme focussing on reducing episodes of hypoglycaemia. At 6 months, IAH had improved in all groups and rates of severe hypoglycaemia fell from 77% of individuals in the 6 months before the trial to 20% during the 6 month trial (pre-HypoCOMPaSS 8.9 ± 13.4 vs post-HypoCOMPaSS 0.8 ± 1.8 episodes per person per year; *p* = 0.0001) without deterioration in HbA_1c_ (pre-HypoCOMPaSS 8.2% [66.1 mmol/mol] vs post-HypoCOMPaSS 8.1% [65 mmol/mol]) [34]. Importantly, neither pumps nor CGM (or a combination of both) produced any major additional positive effect compared with education and ongoing support alone. Since all three arms of this study were delivered in participating sites, there is a possibility of ‘contamination’ between findings. Furthermore, it was not possible to ascertain whether the benefit was due to the half-day education or the weekly support provided by the research fellows. Since it seems unlikely that brief education alone would have caused this degree of behaviour change, it is probable that both components contributed to these outcomes.

### The Blood Glucose Awareness Training programme

Not all individuals respond to structured education and continue to experience IAH and recurrent hypoglycaemia. Some have psychological and cognitive barriers that interfere with their ability to avoid hypoglycaemia [35]. A number of interventions have been developed specifically to address the problems associated with IAH. However, not all of these interventions are available for individuals who have previously participated in structured education. The interventions are often described as ‘psycho-behavioural’ but, although some are delivered by psychologists, the psychological theory on which they are based is not always described in detail. Some include elements of structured education while others focus on other aspects of self-management. In the absence of detailed descriptions of the development of these interventions in the published literature, we have found it difficult to identify any common features of these programmes. However, Blood Glucose Awareness Training (BGAT) provides an example of how psychological intervention may be included in an education programme to improve hypoglycaemia awareness.

The BGAT programme uses skills-based training to help participants to detect both hypoglycaemia and hyperglycaemia over an 8 week period [36]. In BGAT, individuals are taught to focus on enhanced awareness of internal cues including physical symptoms (such as motor performance), cognitive skills (such as difficulty in concentrating) and mood changes. Participants in BGAT programmes are taught to use these signals to identify subtle variations in blood glucose. They are also taught to recognise external cues, including timing and dose of previous insulin, food consumption and physical exercise, to anticipate changes in blood glucose. The programme appears to recruit some individuals with no previous experience of structured education. A trained psychologist assesses progress and provides maintenance strategies. In both outpatient [37] and inpatient [38] settings, BGAT has successfully promoted improved detection of hypoglycaemia in those with IAH and fewer low glucose readings in those with awareness of hypoglycaemia, without deteriorating glycaemic control.

A limitation of BGAT studies [36, 39] is baseline rates of severe hypoglycaemia before intervention were not reported. Moreover, in the study by Kinsley et al [36], participants that had experienced an episode of severe hypoglycaemia in the preceding 2 years prior to the study were excluded, providing a potential selection bias. Conversely, in a Swiss study using BGAT principles, those with recurrent severe hypoglycaemia were specifically encouraged to participate and a demonstrable decline in severe hypoglycaemia rates (nearly 90%) in the education arm was observed [40]. Kinsley et al measured counter-regulatory responses to hypoglycaemia, which were improved in those randomised to BGAT compared with control participants. This study also showed an improvement in glycaemic control, which was not observed in the Swiss BGAT trial [36, 40].

### The Hypoglycemia Anticipation, Awareness and Treatment Training programme

Like BGAT, studies into Hypoglycemia Anticipation, Awareness and Treatment Training (HAATT) have demonstrated that structured education concentrating on hypoglycaemia detection in type 1 diabetes can reduce rates of moderate hypoglycaemia and severe hypoglycaemia (pre-HAATT 2.0 vs post HAATT 0.4 episodes per person per year; *p* < 0.05) without compromising metabolic control (pre-HAATT HbA_1c_ 8.1% [65 mmol/mol] vs post-HAATT 8.0% [63.9 mmol/mol]) [41]. The psychological element of HAATT focussed on devising individual plans for maintaining HAATT principles following the course.

### HyPOS

A structured education programme developed by Hermanns and colleagues in Germany [42] involves five 90 min sessions delivered to groups of individuals with IAH over 5 weeks. This intervention, known as HyPOS, differed from other studies by exclusively focussing on hypoglycaemia as opposed to broader principles of diabetes self-management. When compared with a control group (who received standard group education) at 6 months, awareness of hypoglycaemia had improved in the intervention group, although rates of severe hypoglycaemia were not different. Interestingly, when 140 of the original 164 participants were evaluated at 31 months, rates of severe hypoglycaemia in the intervention group were around half of those in the control group (mean ± SD: 0.1 ± 0.2 vs 0.2 ± 0.4 episodes per person per year; *p* = 0.04) [43] . However, loss to follow-up was not reported and, for reasons which were unclear, rates of severe hypoglycaemia in both arms had fallen by fourfold compared with baseline.

### DAFNE-Hypoglycaemia Awareness Restoration Training

The DAFNE-Hypoglycaemia Awareness Restoration Training (DAFNE-HART) pilot study is a psychoeducational intervention aimed for individuals who experience IAH following DAFNE training [44]. The DAFNE-HART study recruited 24 people with type 1 diabetes that had IAH assessed clinically as a Gold score ≥ 4. Participants were encouraged to seek hypoglycaemia cues and consider consequences of hypoglycaemia and IAH using motivational interviewing and cognitive behavioural therapy techniques, delivered over 6 weeks by DAFNE educators trained by a clinical psychologist. In the pilot trial, the investigators reported a significant reduction in the incidence of severe hypoglycaemia (median [range]: pre-DAFNE-HART three [0–104] vs post-DAFNE-HART no [0–3] episodes per person per year; *p* < 0.001), as well as improved awareness of hypoglycaemia. HbA_1c_ was unchanged (pre-DAFNE-HART 7.8% [62 mmol/mol] vs post-DAFNE-HART 7.8% [61.8 mmol/mol]) 12 months post intervention [44].

The DAFNE-HART study was an uncontrolled small-scale pilot study. However, it appears to be the first intervention to recruit individuals with previous experience of structured education but who continued to have problems with hypoglycaemia. The study suggests that targeting unhelpful cognitions and beliefs may be particularly effective in some individuals although this needs to be confirmed by an adequately powered RCT.
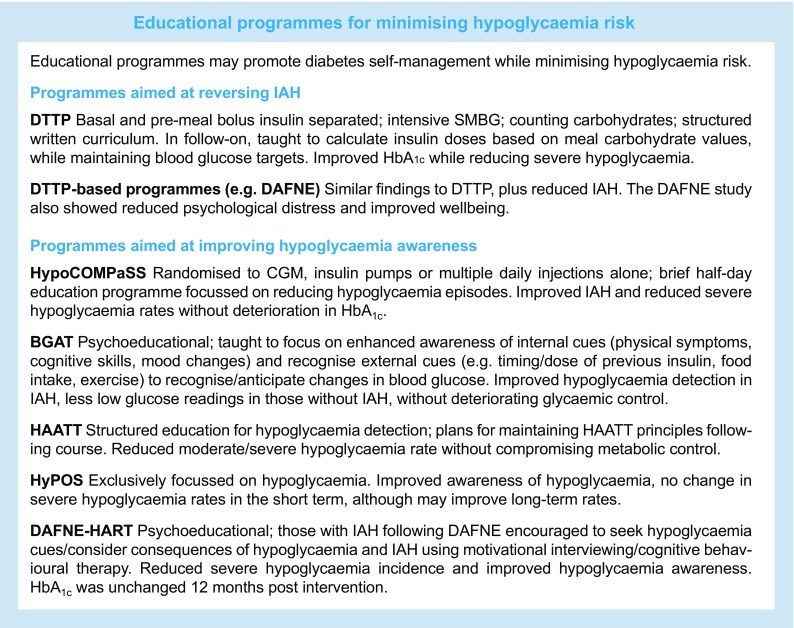


### Comparison of programmes to improve hypoglycaemia awareness

A recent systematic review and meta-analysis examining interventions that restore awareness of hypoglycaemia in type 1 diabetes identified 27 studies that used structured education as the primary intervention [9]. Of these, only six studies were RCTs (Table 1) and the number of individuals enrolled was generally less than 200 in each; the remaining studies were retrospective and observational. Of these clinical trials, three studies recruited individuals who had type 1 diabetes and had IAH [41–43], while the other four recruited unselected individuals [36, 39, 40, 45]. When comparing head-to-head educational programmes that incorporate a psychological approach vs those that focus primarily on structured education of insulin therapy, no significant difference in severe hypoglycaemia has been observed [9]. However, the relatively few adequately powered RCTs in this area makes it difficult to establish the benefit of structured educational interventions. Furthermore, it is clearly impossible to blind clinical trials of a behavioural intervention although recording rates of severe hypoglycaemia (if reported accurately) and awareness scores are reasonably objective.

**Table 1:** Summary of key RCTs of structured education programmes in the management of type 1 diabetes

Study	Educational intervention	Participants in study arms, *n*	Age, years; diabetes duration, mean years ± SD	Inclusion of those with IAH	Inclusion of those with SH in the year before intervention	Impact on hypoglycaemia detection/awareness	Impact on SH incidence, %	HbA_1c_ change pre- vs post-intervention, % (mmol/mol)	Duration of follow-up
Cox et al (1994) [39]	Long-term follow-up evaluation of individuals receiving BGAT in two separate previous studies	Total, 41; BGAT, 14; BGAT + booster training, 14; control, 13	42.9 ± 3.5; 16.3 ± 2.8	Unselected inclusion of individuals with T1D; those with IAH not excluded	Yes	Hypoglycaemia (< 2.8 mmol/l) detection rate: BGAT, 50%; BGAT + booster, 85%; control, 43% (*p* < 0.02 control vs BGAT and BGAT + booster)	SH rate not reported	BGAT, 12.3 (110.9) vs 10.2 (88); control 11.4 (101.1)vs 9.9 (84.7) (NS)	4.9 years
Kinsley et al (1999) [36]	BGAT vs control group (cholesterol awareness) training	Total, 47; BGAT, 25; control, 22	34.8 ± 8; 9 ± 3	Unselected inclusion of individuals with T1D, those with IAH not excluded	No	Increased adrenaline (epinephrine) response to hypoglycaemia in BGAT group (*p* < 0.05); increased frequency of hypoglycaemia (<3.9 mmol/l) in BGAT and control group (NS)	SH rate not reported	BGAT, 9.0 (74.9) vs 7.8 (61.7); control, 9.1 (76) vs 7.9 (62.8) (NS)	4 months
Cox et al (2004) [41]	HAATT (7-week psycho-educational intervention based on BGAT) vs control group (SMBG)	Total, 40; HAATT, 20; control, 20	38.1 ± 9.3; 13.9 ± 8.5	Yes	Yes	Improved detection of hypoglycaemia (<3.9 mmol/l) in HAATT (*p* = 0.005): pre- vs post-intervention, 52% vs 70%; pre- vs post-control, 58% to 55%	Reduced SH in HAATT (*p* = 0.03): pre- vs post-intervention, 2.0 vs 0.4 episodes per person per year; pre- vs post-control, 1.8 vs 1.7 episodes per person per year	HAATT, 8.1 (65) vs 9.0 (74.9); control, 8.0 (63.9) vs 8.1 (65.0) (NS)	18 months
Schachinger et al (2005) [40]	BGAT (III) (Swiss study) vs control group	Total, 111; BGAT, 56; control, 55	46.4 ± 13.8; 22.9 ± 12.1	Unselected inclusion of individuals with T1D, those with IAH not excluded	Yes	Improved detection of hypoglycaemia in BGAT (III) (*p* = 0.005): pre- vs post-intervention, 52.7% vs 65.2%; pre- vs post-control, 53.5% vs 48%	Reduced SH in BGATT (III) (*p* < 0.05): pre- vs post-intervention, 1.61 vs 0.33 episodes per person per year; pre- vs post-control, 1.76 vs 1.78 episodes per person per year	No change: mean 6.9 (51.9) in BGAT(III) and control group	12 months
Hermanns et al (2010) [43]	HyPOS intervention vs control group (standard education). Long-term follow-up of the original HyPOS intervention [42]	Total, 64; HyPOS, 84; control, 80	46 ± 12.5; 21.4 ± 10.9	Yes	Yes	Not reported (in original study [39], improvement in the modified Gold score [0–10] [*p* = 0.015]; pre- vs post-HyPOS, 4.3 vs 6.1; pre- vs post-control, 4.3 vs 5.3)	Reduced SH in HyPOS (*p* = 0.04): HyPOS, 0.1 episodes per person per year vs control, 0.2 episodes per person per year	No change: mean 7.2 (55.2) in HyPOS and control group	31 months
Hermanns et al (2013) [45]	Novel PRIMAS intervention vs control group (undergoing DTTP)	Total, 160; PRIMAS, 81; control, 79	45.4 ± 13.6; 19.5 ± 13	Unselected inclusion of individuals with T1D, those with IAH not excluded	Yes	Improvement in hypoglycaemia awareness according to modifiedClarke score^a^ in both groups (NS):pre- vs post-PRIMAS, 1.5 vs 1.2; pre- vs post-control, 1.8 vs 1.3	Reduced SH in both PRIMAS and control groups (NS): pre- vs post-PRIMAS, 0.29 vs 0.06 episodes per person per year; pre- vs post-control, 0.31 to 0.01 episodes per person per year	Pre- vs post-PRIMAS, 8.3 (67.2) vs 7.9 (62.8); pre- vs post-control, 8.1% (65) (*p* = 0.012 between groups)	6 months

^a^Scale ranges from 0 (full awareness of hypoglycaemia) to 7 (complete hypoglycaemia unawareness), where a score of ≥4 suggests impaired awareness of hypoglycaemia.

NS, non-significant difference between groups; PRIMAS, programme for diabetes education and treatment for a self-determined living with type 1 diabetes; SH, severe hypoglycaemia; T1D, type 1 diabetes

### Summary of findings

In summary, evidence from trial data suggests that structured education reduces the incidence of severe hypoglycaemia and improves awareness of hypoglycaemia in those with IAH in type 1 diabetes. Longer-term observational follow-up indicates a reduction in the rate of severe hypoglycaemia of around 50% following educational interventions. This is comparable with the reduction seen with technological interventions including CSII, although one recent trial involving insulin suspend pumps has reported larger reductions in severe hypoglycaemia rates [46]. Another recent RCT studying those with type 1 diabetes and IAH reported that CGM reduced severe hypoglycaemia rates by approximately 40% vs SMBG [47]. The authors controlled for education by re-training participants in both arms on diabetes management prior to randomisation.

High-quality RCTs investigating the role of structured education in those with insulin-treated type 2 diabetes are generally lacking and even fewer have reported rates of severe hypoglycaemia. A group from Korea have evaluated a hypoglycaemia reduction course following structured intensive diabetes education for adults with type 2 diabetes treated with insulin or sulfonylureas [48]. They randomised 28 of 56 individuals who had received the initial course to receive additional reinforcement teaching on hypoglycaemia. Over 6 months, the intervention group experienced half as many symptomatic episodes. In contrast, a Chinese study found no differences in rates of symptomatic hypoglycaemia between adults with type 2 diabetes starting insulin therapy who were randomised to receive structured education and their control group [49]. The X-PERT programme, delivered in the UK, studied 314 people with type 2 diabetes and randomised them to either individual appointments with a dietitian (control group) or a self-management programme administered over six 2 h visits with a dietitian (intervention group) [50]. There was a significant reduction in mean HbA_1c_ (0.6%) in addition to improved body weight, lipid profiles, foot care and overall satisfaction in the intervention group vs control group [50]. There was, however, no statistically significant difference in the mean frequency of hypoglycaemia between the intervention and control groups at 4 month follow-up. It appears that only a small proportion (<20%) were on insulin at baseline.

In light of the rising global costs associated with diabetes care, recently estimated at a substantial 825 billion US dollars annually [51], it is relevant to compare the cost-effectiveness of educational interventions in relation to other approaches, such as technology. Economic modelling of the DAFNE programme indicates greater life expectancy and reduced incidence of diabetes-related complications and this programme would be considered cost-effective according to the National Institute for Health and Care Excellence (NICE) criteria [52]. Evidence in type 2 diabetes also suggests that structured education, which empowers individuals to self-manage their condition and leads to positive changes in lifestyle, yields added health benefits at reasonable costs [53].

## Conclusions

Despite significant technological advances in insulin therapy over the last 20 years, hypoglycaemia continues to be a major barrier to the effective treatment of diabetes, which aims to prevent the development of diabetes-related complications. There is reasonable evidence from both RCTs and observational studies that structured education (defined as insulin self-management and/or specific training in hypoglycaemia avoidance) leads to reductions in the rates of severe hypoglycaemia in type 1 diabetes, while improving glycaemic control. Emerging evidence suggests that individuals with IAH at high risk of severe hypoglycaemia benefit from specific educational interventions. This particularly applies to structured approaches that target both educational and psychological issues, although further research is needed. No studies in type 2 diabetes have demonstrated that structured education can lower the risk of severe hypoglycaemia in individuals with insulin-treated type 2 diabetes. There is an urgent need for further work, particularly in those at greatest potential risk (i.e. a long duration of insulin treatment and reduced hypoglycaemia awareness). In the meantime, structured education should be part of routine management for all those with type 1 diabetes.

## Electronic supplementary material

ESM Downloadable slide (PPTX 113 kb)

## Abbreviations

BGAT: Blood Glucose Awareness Training
CGM: Continuous glucose monitoring
CSII: Continuous subcutaneous insulin infusion
DAFNE: Dose Adjustment for Normal Eating
DAFNE-HART: DAFNE-Hypoglycaemia Awareness Restoration Training
DTTP: Diabetes Treatment and Teaching programme
HAATT: Hypoglycemia Anticipation, Awareness and Treatment Training
HypoCOMPaSS: Recovery of Hypoglycemia Awareness in Long-Standing Type 1 Diabetes
IAH: Impaired awareness of hypoglycaemia
SMBG: Self-monitoring of blood glucose
TIM: Tayside insulin management

## References

[1] The Diabetes Control and Complications Trial Research Group (1993). The effect of intensive treatment of diabetes on the development and progression of long-term complications in insulin-dependent diabetes mellitus. N Engl J Med.

[2] UK Prospective Diabetes Study (UKPDS) Group (1998). Intensive blood-glucose control with sulphonylureas or insulin compared with conventional treatment and risk of complications in patients with type 2 diabetes (UKPDS 33). Lancet.

[3] Bergenstal RM, Tamborlane WV, Ahmann A (2010). Effectiveness of sensor-augmented insulin-pump therapy in type 1 diabetes. N Engl J Med.

[4] Tamborlane WV, Beck RW, Bode BW (2008). Continuous glucose monitoring and intensive treatment of type 1 diabetes. N Engl J Med.

[5] UK Hypoglycaemia Study Group (2007). Risk of hypoglycaemia in types 1 and 2 diabetes: effects of treatment modalities and their duration. Diabetologia.

[6] Kristensen PL, Hansen LS, Jespersen MJ (2012). Insulin analogues and severe hypoglycaemia in type 1 diabetes. Diabetes Res Clin Pract.

[7] Khunti K, Alsifri S, Aronson R (2016). Rates and predictors of hypoglycaemia in 27 585 people from 24 countries with insulin-treated type 1 and type 2 diabetes: the global HAT study. Diabetes Obes Metab.

[8] MacLeod KM, Hepburn DA, Frier BM (1993). Frequency and morbidity of severe hypoglycaemia in insulin-treated diabetic patients. Diabet Med.

[9] Yeoh E, Choudhary P, Nwokolo M, Ayis S, Amiel SA (2015). Interventions that restore awareness of hypoglycemia in adults with type 1 diabetes: a systematic review and meta-analysis. Diabetes Care.

[10] American Diabetes Association Workgroup on Hypoglycemia (2005). Defining and reporting hypoglycemia in diabetes. Diabetes Care.

[11] The International Hypoglycaemia Study Group (2017) Glucose concentrations of less than 3.0 mmol/l (54 mg/dl) should be reported in clinical trials: a joint position statement of the American Diabetes Association and the European Association for the Study of Diabetes. Diabetologia 60:3–6 10.1007/s00125-016-4146-6 PMC6518070 27872948

[12] Jaap AJ, Jones GC, McCrimmon RJ, Deary IJ, Frier BM (1998). Perceived symptoms of hypoglycaemia in elderly type 2 diabetic patients treated with insulin. Diabet Med.

[13] Donnelly LA, Morris AD, Frier BM (2005). Frequency and predictors of hypoglycaemia in type 1 and insulin-treated type 2 diabetes: a population-based study. Diabet Med.

[14] Pramming S, Thorsteinsson B, Bendtson I, Binder C (1991). Symptomatic hypoglycaemia in 411 type 1 diabetic patients. Diabet Med.

[15] Hepburn DA, Patrick AW, Eadington DW, Ewing DJ, Frier BM (1990). Unawareness of hypoglycaemia in insulin-treated diabetic patients: prevalence and relationship to autonomic neuropathy. Diabet Med.

[16] Muhlhauser I, Berger M, Sonnenberg G (1985). Incidence and management of severe hypoglycemia in 434 adults with insulin-dependent diabetes mellitus. Diabetes Care.

[17] Schopman JE, Geddes J, Frier BM (2010). Prevalence of impaired awareness of hypoglycaemia and frequency of hypoglycaemia in insulin-treated type 2 diabetes. Diabetes Res Clin Pract.

[18] Hepburn DA, MacLeod KM, Pell AC, Scougal IJ, Frier BM (1993). Frequency and symptoms of hypoglycaemia experienced by patients with type 2 diabetes treated with insulin. Diabet Med.

[19] Hossain P, Kawar B, El Nahas M (2007). Obesity and diabetes in the developing world—a growing challenge. N Engl J Med.

[20] Fanelli CG, Epifano L, Rambotti AM (1993). Meticulous prevention of hypoglycemia normalizes the glycemic thresholds and magnitude of most of neuroendocrine responses to, symptoms of, and cognitive function during hypoglycemia in intensively treated patients with short-term IDDM. Diabetes.

[21] Cranston I, Lomas J, Maran A, Macdonald I, Amiel SA (1994). Restoration of hypoglycaemia awareness in patients with long-duration insulin-dependent diabetes. Lancet.

[22] Heller SR, Cryer PE (1991). Reduced neuroendocrine and symptomatic responses to subsequent hypoglycemia after 1 episode of hypoglycemia in nondiabetic humans. Diabetes.

[23] Dagogo-Jack SE, Craft S, Cryer PE (1993). Hypoglycemia-associated autonomic failure in insulin-dependent diabetes mellitus. Recent antecedent hypoglycemia reduces autonomic responses to, symptoms of, and defense against subsequent hypoglycemia. J Clin Invest.

[24] Muhlhauser I, Jorgens V, Berger M (1983). Bicentric evaluation of a teaching and treatment programme for type 1 (insulin-dependent) diabetic patients: improvement of metabolic control and other measures of diabetes care for up to 22 months. Diabetologia.

[25] Mühlhauser I, Bruckner I, Berger M (1987). Evaluation of an intensified insulin treatment and teaching programme as routine management of type 1 (insulin-dependent) diabetes. Diabetologia.

[26] Starostina EG, Antsiferov M, Galstyan GR (1994). Effectiveness and cost-benefit analysis of intensive treatment and teaching programmes for type 1 (insulin-dependent) diabetes mellitus in Moscow—blood glucose versus urine glucose self-monitoring. Diabetologia.

[27] Bott S, Bott U, Berger M, Muhlhauser I (1997). Intensified insulin therapy and the risk of severe hypoglycaemia. Diabetologia.

[28] Sämann A, Mühlhauser I, Bender R, Kloos C, Müller UA (2005). Glycaemic control and severe hypoglycaemia following training in flexible, intensive insulin therapy to enable dietary freedom in people with type 1 diabetes: a prospective implementation study. Diabetologia.

[29] Plank J, Kohler G, Rakovac I (2004). Long-term evaluation of a structured outpatient education programme for intensified insulin therapy in patients with type 1 diabetes: a 12-year follow-up. Diabetologia.

[30] DAFNE Study Group (2002). Training in flexible, intensive insulin management to enable dietary freedom in people with type 1 diabetes: dose adjustment for normal eating (DAFNE) randomised controlled trial. BMJ.

[31] Hopkins D, Lawrence I, Mansell P (2012). Improved biomedical and psychological outcomes 1 year after structured education in flexible insulin therapy for people with type 1 diabetes: the U.K. DAFNE experience. Diabetes Care.

[32] Jordan LV, Robertson M, Grant L (2013). The Tayside insulin management course: an effective education programme in type 1 diabetes. Int J Clin Pract.

[33] Gold AE, Macleod KM, Frier BM (1994). Frequency of severe hypoglycemia in patients with type I diabetes with impaired awareness of hypoglycemia. Diabetes Care.

[34] Little SA, Leelarathna L, Walkinshaw E (2014). Recovery of hypoglycemia awareness in long-standing type 1 diabetes: a multicenter 2 x 2 factorial randomized controlled trial comparing insulin pump with multiple daily injections and continuous with conventional glucose self-monitoring (HypoCOMPaSS). Diabetes Care.

[35] Rogers HA, de Zoysa N, Amiel SA (2012). Patient experience of hypoglycaemia unawareness in type 1 diabetes: are patients appropriately concerned?. Diabet Med.

[36] Kinsley BT, Weinger K, Bajaj M (1999). Blood glucose awareness training and epinephrine responses to hypoglycemia during intensive treatment in type 1 diabetes. Diabetes Care.

[37] Cox DJ, Gonder-Frederick LA, Lee JH, Julian DM, Carter WR, Clarke WL (1989). Effects and correlates of blood glucose awareness training among patients with IDDM. Diabetes Care.

[38] Cox D, Gonder-Frederick L, Polonsky W, Schlundt D, Julian D, Clarke W (1995). A multicenter evaluation of blood glucose awareness training-II. Diabetes Care.

[39] Cox DJ, Gonder-Frederick L, Julian DM, Clarke W (1994). Long-term follow-up evaluation of blood glucose awareness training. Diabetes Care.

[40] Schachinger H, Hegar K, Hermanns N (2005). Randomized controlled clinical trial of blood glucose awareness training (BGAT III) in Switzerland and Germany. J Behav Med.

[41] Cox DJ, Kovatchev B, Koev D (2004). Hypoglycemia anticipation, awareness and treatment training (HAATT) reduces occurrence of severe hypoglycemia among adults with type 1 diabetes mellitus. Int J Behav Med.

[42] Hermanns N, Kulzer B, Kubiak T, Krichbaum M, Haak T (2007). The effect of an education programme (HyPOS) to treat hypoglycaemia problems in patients with type 1 diabetes. Diabetes Metab Res Rev.

[43] Hermanns N, Kulzer B, Krichbaum M, Kubiak T, Haak T (2010). Long-term effect of an education program (HyPOS) on the incidence of severe hypoglycemia in patients with type 1 diabetes. Diabetes Care.

[44] de Zoysa N, Rogers H, Stadler M (2014). A psychoeducational program to restore hypoglycemia awareness: the DAFNE-HART pilot study. Diabetes Care.

[45] Hermanns N, Kulzer B, Ehrmann D, Bergis-Jurgan N, Haak T (2013). The effect of a diabetes education programme (PRIMAS) for people with type 1 diabetes: results of a randomized trial. Diabetes Res Clin Pract.

[46] Ly TT, Nicholas JA, Retterath A, Lim EM, Davis EA, Jones TW (2013). Effect of sensor-augmented insulin pump therapy and automated insulin suspension vs standard insulin pump therapy on hypoglycemia in patients with type 1 diabetes: a randomized clinical trial. JAMA.

[47] van Beers CA, DeVries JH, Kleijer SJ (2016). Continuous glucose monitoring for patients with type 1 diabetes and impaired awareness of hypoglycaemia (IN CONTROL): a randomised, open-label, crossover trial. Lancet Diabetes Endocrinol.

[48] Yong YM, Shin KM, Lee KM (2015). Intensive individualized reinforcement education is important for the prevention of hypoglycemia in patients with type 2 diabetes. Diabetes Metab J.

[49] Guo XH, Ji LN, Lu JM (2014). Efficacy of structured education in patients with type 2 diabetes mellitus receiving insulin treatment. J Diabetes.

[50] Deakin TA, Cade JE, Williams R, Greenwood DC (2006). Structured patient education: the diabetes X-PERT Programme makes a difference. Diabet Med.

[51] NCD Risk Factor Collaboration (NCD-RisC) (2016). Worldwide trends in diabetes since 1980: a pooled analysis of 751 population-based studies with 4.4 million participants. Lancet.

[52] Kruger J, Brennan A, Thokala P (2013). The cost-effectiveness of the dose adjustment for normal eating (DAFNE) structured education programme: an update using the Sheffield type 1 diabetes policy model. Diabet Med.

[53] Jacobs-van der Bruggen MA, van Baal PH, Hoogenveen RT (2009). Cost-effectiveness of lifestyle modification in diabetic patients. Diabetes Care.

