# Supplementary material for: The role of structured education in the management of hypoglycaemia
Source: Diabetologia. 2017 Jun 28;61(4):751–60. doi: 10.1007/s00125-017-4334-z (PMC6448987; doi:10.1007/s00125-017-4334-z)
Supplement: Supplementary file 1 — (PPTX 113 kb) [file 125_2017_4334_MOESM1_ESM.pptx]

## Slide 1
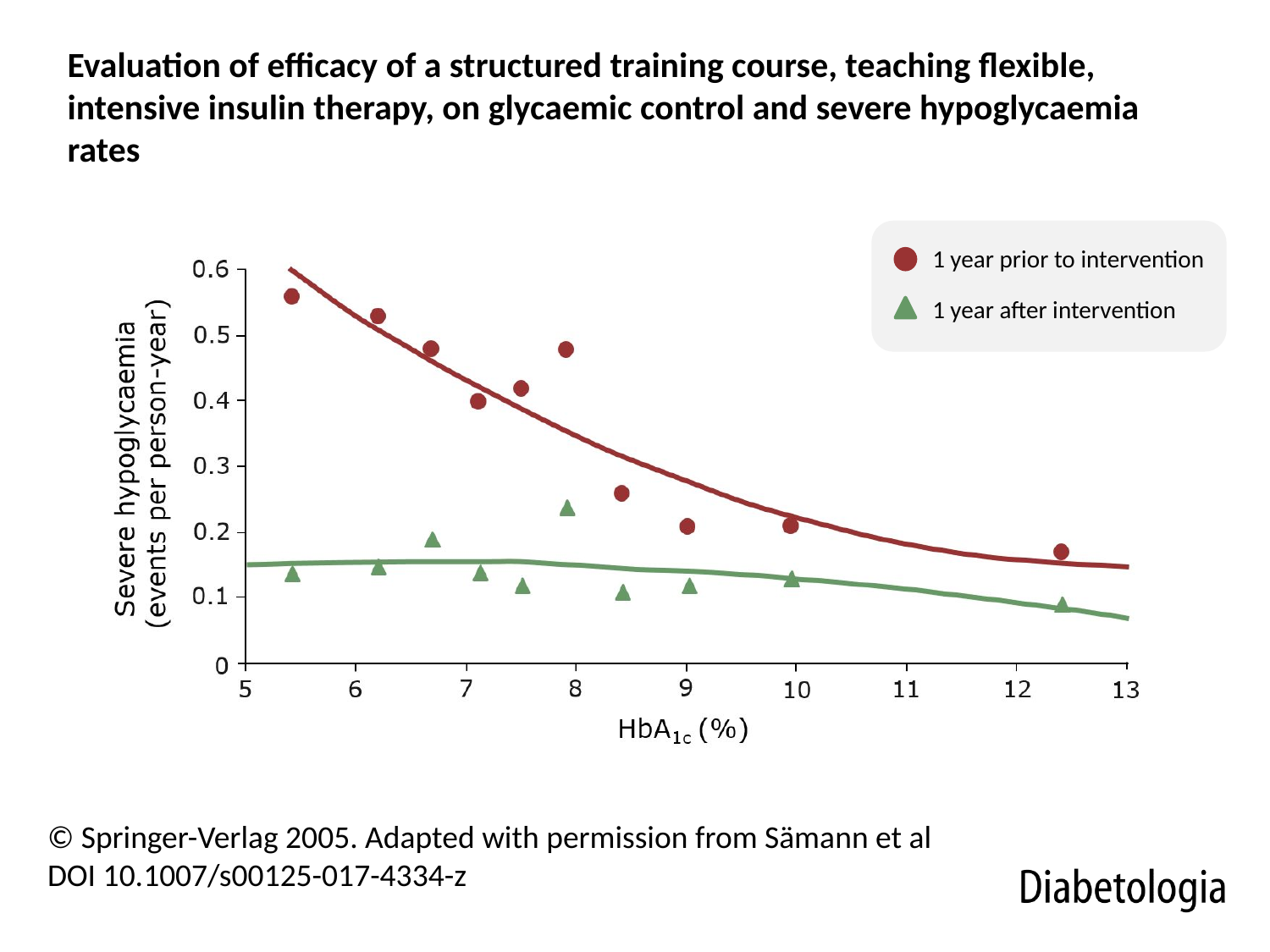

Evaluation of efficacy of a structured training course, teaching flexible, intensive insulin therapy, on glycaemic control and severe hypoglycaemia rates
1 year prior to intervention
1 year after intervention
© Springer-Verlag 2005. Adapted with permission from Sämann et al
DOI 10.1007/s00125-017-4334-z
